# Lungs exposed to 1 hour warm ischemia without heparin before harvesting might be suitable candidates for transplantation

**DOI:** 10.1186/s13019-015-0339-1

**Published:** 2015-10-23

**Authors:** Annika Liersch-Nordqvist, Richard Ingemansson, Leif Pierre, Joanna Hlebowicz, Sandra Lindstedt

**Affiliations:** 1 Department of Pediatric Anesthesia and Intensive Care, Skåne University Hospital, Lund University, Lund, Sweden; 2 Department of Cardiothoracic Surgery, Skåne University Hospital, Lund University, Lund, Sweden; 3 Department of Medicine, Skåne University Hospital, Lund University, Malmö, Sweden

**Keywords:** Lung transplantation, Donation after cardiac death, Ex vivo lung perfusion, Experimental animal study, Heart beating donor

## Abstract

**Background:**

The limiting factor for lung transplantation is the lack of donor organs. The usage of lungs from donation after cardiac death (DCD) would dramatically increase donor availability. In the present paper we wanted to investigate lungs exposed to 1 h of warm ischemia without heparin followed by flush-perfusion and cold storage compared to lungs harvested from heart beating donors (HBD) using standard harvesting technique.

**Methods:**

Twelve Swedish domestic pigs were randomized into two groups. Six pigs (DCD group) underwent ventricular fibrillation and were then left untouched for 1 h after declaration of death. They did not receive heparin. The lungs were then harvested and flush-perfused with Perfadex® solution and the organs were stored at 8 °C for 4 h. Six pigs (HBD group) received heparin and the lungs were harvested and flush-perfused with Perfadex® solution and the organs were stored at 8 °C for 4 h. Lung function was evaluated, using ex vivo lung perfusion (EVLP), with blood gases at different oxygen levels, pulmonary vascular resistance (PVR), lung weight, and macroscopic appearance.

**Results:**

At FiO_2_ 1.0, the PaO_2_ in the DCD group was 51.7 ± 2.0 kPa and in the HBD group 68.6 ± 2.4 kPa (*p* < 0.01). Significantly lower PVR levels were measured in the DCD group (372 ± 31 dyne x s/cm^5^) compared to the HBD group (655 ± 45 dyne x s/cm^5^) (*p* < 0.001). There was no significant difference between groups in weight, compliance or signs of pulmonary thrombosis or embolization.

**Conclusions:**

It seems as if DCD lungs exposed to 1 h of warm ischemia before 4 h of cold storage has satisfying oxygenation capacity, low PVR, normal weight and no signs of thrombosis or embolization. According to our study it seems as lungs exposed to 1 h warm ischemia without heparin might be good candidates for transplantation.

## Background

The shortage of suitable organ donors is still a limiting factor for lung transplantation. Only about 20 % of suitable donor lungs are being transplanted [1, 2]. This leaves a growing number of patients with end-stage pulmonary disease remaining indefinitely on the waiting list for lung transplantation. In recent years, the use of donation after cardiac death (DCD) donors for lung transplantation has grown in interest and is entering clinical practice [3–5]. To be able to evaluate the donor lungs from DCD, the ex vivo lung evaluation (EVLP) method was developed and established by Steen and colleagues at our clinic at the Skane University hospital in Sweden. The EVLP method is today mainly used for lung evaluation in donor lungs from heart beating donors (HBD), where the lungs have initially been rejected prior to eventual clinical lung transplantation at many cardiothoracic clinics all over the world [6–12].

The increasing interest in DCD to increase donor organs has led to extensive research in the field of EVLP and the ideal preservation method. There are reports demonstrating that a time frame of 60 min of warm ischemia does not seem to compromise the pulmonary graft [13–15]. In these publications all animals received heparin before being exposed to the warm ischemia.

In clinical lung transplantation today the donor receives intravenous heparin prior to lung harvesting to avoid lung thrombosis in the lung grafts. In DCD lungs, heparin would need to be recirculated. It is currently being debated whether it is ethically and legal permissible to give a patient heparin after death has been declared but before permission for donation has been received, particularly given the cardiac compressions required to circulate heparin. The avoidance of heparin would help overcome this ethical challenge.

In the present study we investigate the use of heparin infusion prior to lung harvesting using standard clinical routine in a HBD setting vs no heparin in a DCD setting with 1 h warm ischemia before harvesting. Both groups of lungs were flush-perfused with Perfadex® solution and stored for 4 h at 8 °C before being evaluated in the EVLP system. Our intensions were to investigate if DCD lungs without heparin is good enough to transplant regarding blood gases, weight, compliance, thromboembolism and macroscopic appearance. The HBD group was used as a golden standard control.

## Method

### Animal preparation

Twelve Swedish landrace pigs were fasted overnight with free access to water. The experimental protocol for this study was approved by the Ethics Committee for Animal Research, Lund University, Sweden, Dnr M 172-11. All animals received care according to the European Convention of the Protection of Vertebrate Animals used for Experimental and Other Scientific Purposes, the National Society for Medical Research’s Principles of Laboratory Animal Care, and the Institute of Laboratory Animal Research’s Guide for the Care and Use of Laboratory Animals. The experiments described in the manuscript was a part of a larger project setup with many different experimental groups.

The pigs were randomly assigned into 2 groups: the HBD and the DCD group, each group consisting of 6 pigs. Premedication was performed with an intramuscular injection of Xylazine (Rompun® vet. 20 mg/ml; Bayer AG, Leverkusen, Germany; 2 mg/kg) mixed with ketamine (Ketaminol® vet. 100 mg/ml; Farmaceutici Gellini S.p.A., Aprilia, Italy; 20 mg/kg) in their stables, and a peripheral iv access was established in the earlobe. The pig was then transferred to the laboratory and placed in supine position on the operating table. Oral intubation was performed using a 7.5 size endotracheal tube after anesthesia induction with sodium thiopental (Pentothal; Abbott Laboratories, North Chicago, Illinois, USA) and pancuronium bromide (Pavulon; N.V. Organon, Oss, the Netherlands). Anesthesia was maintained with a ketamine (Ketaminol® vet), midazolam (Midazolam Panpharma®, Oslo, Norway), and fentanyl (Leptanal®, Lilly, France) infusion. Fluid loss was compensated for by continuous infusion of Ringer’s Acetate. Mechanical ventilation was established with a Siemens-Elema ventilator (Servo Ventilator 300, Siemens, Solna, Sweden) with an inspired oxygen fraction (FiO_2_) of 0.5, a frequency of 15 breaths/min, a minute ventilation of 6 l/min, and a positive end-exspiratory pressure (PEEP) of 5 cmH_2_O.

### Experimental timeline

**Fig. 1:**
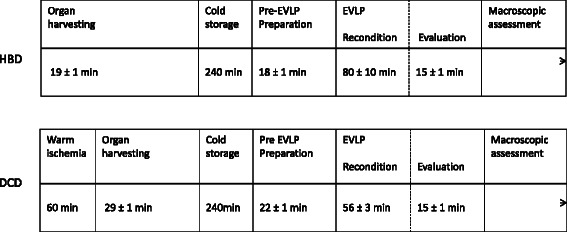
The figure shows a timeline of the experimental setup for the two groups: Heart Beating Donor (HBD) and Donation after Cardiac Death (DCD). The time for each procedural step is given as mean ± SEM. Ex Vivo Lung Perfusion is mentioned as EVLP in the timeline

The experimental timeline is demonstrated in Fig. 1.

### Preservation of HBD Lungs

A median sternotomy was performed. Heparin sodium (Heparin LEO; 400 IE/kg, LEO Pharma AB, Malmö, Sweden,) was given intravenously. The pulmonary artery was cannulated via the right ventricle with a 28 F cannula secured with a purse string suture placed in the outflow tract of the pulmonary artery. A clamp was put on the superior vena cava, and another clamp was put on the inferior vena cava. A clamp was then put on the ascending aorta. The left atrium and inferior vena cava was opened. Ice slush was put in the right and left pleura for cooling of the lungs. The lungs were perfused antegradely with 4 L of cold Perfadex® solution with added isotonic trometamol 1.0 ml (Addex-THAM 3.3 mmol/ml; Fresenius Kabi AB Uppsala, Sweden), calcium chloride 2 ml (0.45 mmol/ml) and nitro-glycerine 3 ml (5 mg/ml; BMM Pharma AB, Stockholm, Sweden) distributed at low perfusion pressure (<20 mmHg). The cannula was removed from the pulmonary artery. The lungs were harvested *en bloc* in a standard fashion. After harvesting, the lungs were put on a scales and the lung weight was noted. During retrieval, a segment (~8 cm) of the descending aorta was also excised. The lungs were immersed in cold Perfadex® solution with the aortic segment and put in cold storage at 8 °C for 4 h.

### Preservation of DCD lungs

A median sternotomy was performed. Ventricular fibrillation was induced electrically. The tracheal tube was disconnected from the ventilator when circulatory arrest was confirmed and left open to air. After one hour after the declaration of death the median sternotomy was opened. The pulmonary artery was cannulated via the right ventricle with a 28 F cannula secured with a purse string suture placed in the outflow tract of the pulmonary artery. A clamp was put on the superior vena cava, and another clamp was put on the inferior vena cava. A clamp was put on the ascending aorta. The left atrium and inferior vena cava was opened.

The lungs were perfused antegradely with 4 L of cold Perfadex® solution with added isotonic trometamol 1.0 ml (Addex-THAM 3.3 mmol/ml; Fresenius Kabi AB Uppsala, Sweden), calcium chloride 2 ml (0.45 mmol/ml) and nitro-glycerine 3 ml (5 mg/ml; BMM Pharma AB, Stockholm, Sweden) distributed at low perfusion pressure (< 20 mmHg).

The cannula was removed from the pulmonary artery. The lungs were harvested *en bloc* in a standard fashion. After harvesting, the lungs were put on a scale and the lung weight was noted. During the retrieval, a segment (~8 cm) of the descending aorta was also excised. The lungs were immersed in cold Perfadex® solution with the aortic segment and put in cold storage at 8 °C for 4 h.

### Ex vivo lung perfusion

Ex vivo lung perfusion (EVLP) was performed using the extracorporeal perfusion circuit by Medtronics (Medtronic AB, Kerkrade, the Netherlands; Ex Vivo Lung Evaluation Set).

The system was primed with albumin (500 ml, 50 g/l and 200 ml 200 g/l; Albumin Baxter, Baxter Medical, Kista, Sweden, and 2 units of autologous blood, earlier withdrawn from each donor. Imipenem (0.5 g; Tienam, Merck Sharp & Dohme, Sollentuna, Sweden), insulin (20 IU; Actrapid; Novo Nordisk, Bagsvaerd, Denmark), and heparin (10,000 IU; Leo Pharma, Malmö, Sweden) were added, and isotonic trometamol (Addex-Tham, Kabi, Sweden) was used to buffer the mixed solution to a temperature adjusted pH of 7.4. Gas was supplied to the membrane oxygenator; first oxygen and CO_2_ during the reconditioning phase, and then 93 % nitrogen and 7 % CO_2_ during the testing phase, creating a normal venous blood gas in the perfusate to the pulmonary artery (i.e., the oxygenator is used to deoxygenate the perfusate). Before the perfusion was started, the pulmonary artery was extended by a segment of the descending aorta to make cannulation easier. The pulmonary artery cannula was then connected to the corresponding tube of the extracorporeal circuit, the air was removed, and the shunt of the circuit was clamped. An endotracheal tube was secured in the trachea with a cotton band and connected to the ventilator. The remnant of the left atrium was left open, prohibiting pulmonary outflow obstruction, and maintaining a constant left atrium pressure around 0 mmHg.

A low-flow perfusion at 25 °C was initiated through the lungs. The lungs were gradually warmed by increasing the temperature of the perfusate. When the temperature reached 32 °C, ventilation was started with a FiO_2_ of 0.5 and a minute volume of 1 L/min, and no positive end-expiratory pressure (PEEP). The pump flow was gradually increased, never allowing the pulmonary arterial pressure to exceed 20 mmHg. With the temperature increase of each 1 °C, ventilation was augmented with a corresponding 1 L minute volume. After 20–30 min, normothermia was reached and positive end-expiratory pressure was added to fully expand the lungs and eliminate atelectasis. Blood gases were analyzed under full ventilation at different inspired oxygen fraction levels. Pulmonary vascular resistance (PVR) was calculated using the formula PVR (dyne*sec/cm^5^) = (80 * (Mean Pulmonary Artery Pressure (PAP) – (Pulmonary Cap Wedge Press (PCW) e.g.Left Atrial Pressure)) / Cardiac Output (C.O) e.g. Pulmonary Artery Flow).

The lungs were then disconnected from the EVLP. A deflation test was performed for a final evaluation of the lungs by disconnecting the lungs from the ventilator at the end of inspiration. The lungs were put onto a scale and weighed. The pulmonary arterial branches were macroscopically studied for thrombotic material by opening the arteries as far distally as possible.

### Calculations and statistics

Descriptive statistics, in the form of the number of experimental animals, mean, and the standard error on the mean (SEM) for the different parameters were analyzed. The descriptive results are presented for the different parameters divided into the different groups (HBD, DCD). Statistically significant difference between the different groups was tested by a non-parametric Kruskal-Wallis-test. Adjustments of the p-value were made according to the Bonferroni correction. All statistical analysis was performed, using SPSS version 20. Significance was defined as: *p* < 0.001 (***), *p* < 0.01 (**), *p* < 0.05 (*), and *p* > 0.05 (not significant, n.s.).

## Results

### Study groups

Animal weights in the groups were as follows: 60.2 ± 0.8 kg in the HBD group and 61.2 ± 1.4 kg in the DCD group.

Pre-operative arterial oxygen partial pressure (PaO_2_) at an FiO_2_ of 0.5 were in the HBD group 30.9 ± 0.7 kPa and in the DCD group 29.1 ± 0.9 kPa. (n.s).

Pre-operative arterial carbondioxide partial pressure (PaCO_2_) at an FiO_2_ of 0.5 were in the HBD group 6.5 ± 0.1 kPa and in the DCD group 6.0 ± 0.2 kPa (n.s.).

The EVLP time was 80 ± 9.7 min in the HBD group, 56 ± 3.3 min for the DCD group.

No anatomical anomalies, signs of infection, or malignancy were found in any of the animals at autopsy.

### Pulmonary gas function

#### Arterial and venous blood gases

Arterial and venous blood gases at FiO_2_ 1.0, 0.5, and 0.21 at the end of EVLP are presented in Table 1. In the HBD group, the PaO_2_ was 68.6 ± 2.4 kPa and in the DCD group, the PaO_2_ was 51.7 ± 2.0 kPa after completed EVLP at FiO_2_ 1.0. (*p* < 0.01).

**Table 1:** Arterial and venous blood gases at FiO2 1.0, 0.5, and 0.21 at the end of EVLP

	HBD	DCD	HBD-DCD
	(Mean + SEM)		(*p*-value)
PaO_2_ (kPa)			
*FiO*_*2*_ *1.0*	68.6 ± 2.44	51.7 ± 2.05	<0. 01
*FiO*_*2*_ *0.5*	28.6 ± 1.89	23.4 ± 0.80	n.s.
*FiO*_*2*_ *0.21*	10.1 ± 0.26	9.0 ± 0.35	n.s.
PaCO_2_ (kPa)			
*FiO*_*2*_ *1.0*	3.3 ± 0.23	3.5 ± 0.09	n.s.
*FiO*_*2*_ *0.5*	3.4 ± 0.27	3.3 ± 0.09	n.s.
*FiO*_*2*_ *0.21*	3.4 ± 0.17	3.6 ± 0.10	n.s.
PvO_2_ (kPa)			
*FiO*_*2*_ *1.0*	6.5 ± 0.13	7.1 ± 0.14	n.s.
*FiO*_*2*_ *0.5*	6.6 ± 0.12	6.9 ± 0.20	n.s.
*FiO*_*2*_ *0.21*	3.7 ± 0.03	5.9 ± 0.40	<0. 001
PvCO_2_ (kPa)			
*FiO*_*2*_ *1.0*	3.9 ± 0.05	3.8 ± 0.09	n.s.
*FiO*_*2*_ *0.5*	3.7 ± 0.17	3.6 ± 0.18	n.s.
*FiO*_*2*_ *0.21*	3.8 ± 0.03	4.1 ± 0.08	n.s.

*FiO*_*2*_ *= Inspired oxygen fraction, PaO*_*2*_ *= arterial oxygen partial pressure, PaCO*_*2*_ *= arterial carbon dioxide partial pressure, PvO*_*2*_ *= venous oxygen partial pressure, PvCO*_*2*_ = venous carbon dioxide partial pressure. HBD = heart-beating-donor group, DCD = donation after cardiac death. Significance was defined as p < 0.05 (*), p < 0.01 (**), p < 0.001 (***) and p > 0.05 (n.s.)

### Hemodynamic data

#### Pulmonary artery flow and pulmonary artery pressure

**Fig. 2:**
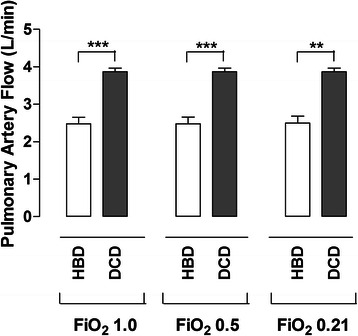
The mean pulmonary artery flow (PAF ± SEM) after Ex Vivo Lung Perfusion (EVLP) is illustrated for the two groups: Heart Beating Donor (HBD) and Donation after Cardiac Death (DCD) at different fractions of inspired oxygen (FiO_2_). Significance was defined as *p* < 0.05 (*), *p* < 0.01 (**), *p* < 0.001 (***) and *p* > 0.05 (n.s.)

The PAF at FiO_2_ 1.0 in the HBD group was 2.5 ± 0.2 L/min and in the DCD group 3.9 ± 0.1 L/min. (*p* < 0.001). PAF at FiO_2_ 0.5 and FiO_2_ 0.21 were identical to PAF values at FiO_2_ 1.0 (Fig. 2).

The PAP at FiO_2_ 1.0 in the HBD group was 19.8 ± 0.2 mmHg and in the DCD group 17.8 ± 1.2 mmHg (n.s). PAP at FiO_2_ 0.5 and FiO_2_ 0.21 were identical to PAP values at FiO_2_ 1.0.

#### Pulmonary vascular resistance

**Fig. 3:**
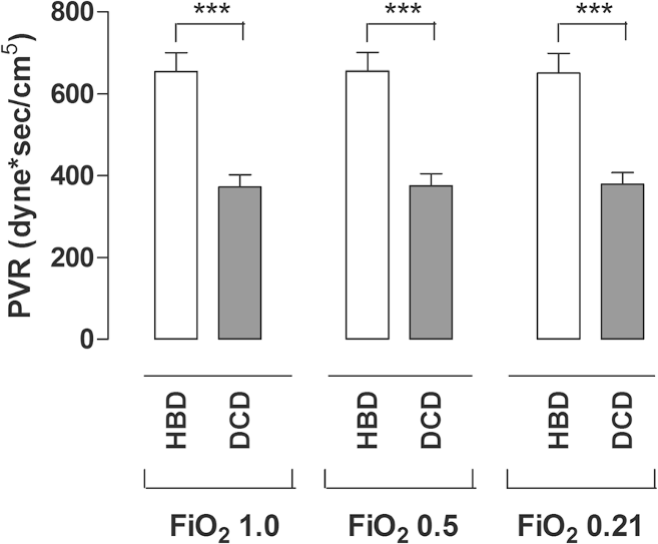
The mean pulmonary vascular resistance (PVR ± SEM) after Ex Vivo Lung Perfusion (EVLP) is illustrated for the two groups: Heart Beating Donor (HBD) and Donation after Cardiac Death (DCD-NH) at different fractions of inspired oxygen (FiO_2_). Significance was defined as *p* < 0.05 (*), *p* < 0.01 (**), *p* < 0.001 (***) and *p* > 0.05 (n.s.)

The PVR was calculated at the different FiO_2_ at 1.0, 0.5, and 0.21 and is also presented in Fig. 3. The PVR at FiO_2_ 1.0 was calculated to 655 ± 45 dyne x s/cm^5^ in the HBD group and in the DCD group PVR was 372 ± 31 dyne x s/cm^5^ (*p* < 0.001).

### Pulmonary graft compliance

After evaluation of the lungs, they were disconnected from the ventilator and a collapse test was performed. If the lungs do not collapse this may indicate lung injury, lung edema, or pneumonia. All the lungs from both study groups collapsed as they should, and showed good compliance. No macroscopically differences between the lungs from the two groups were seen.

### Weight of lungs

The lungs were weighed after harvesting, before EVLP, and after EVLP to assess the degree of lung edema. Before EVLP, the mean lung weight in the HBD group was 510 ± 21 g and in the DCD group 558 ± 21 g (n.s). After EVLP, the lung weight for the HBD group was 513 ± 20 g and for the DCD group 589 ± 24 g (n.s) (Fig. 3).

### Thrombotic material

After completing the lung evaluation, the pulmonary arterial branches were macroscopically studied for thrombotic material by opening the arteries as far distally as possible. No thrombotic material was observed in any of the groups.

## Discussion

With a vast shortage of organs meeting the need for transplantation, the lungs have become a center of focus in potential DCD. The success of Steen and colleagues in transplanting a human uncontrolled DCD lung has led to a new era of DCD lung transplantation [4] even if it is still uncommon to use uncontrolled DCD lungs in clinical practice. Spain is an exception where the extracorporeal circulation setting is part of their protocol in using uncontrolled donors. This has led to the Maastricht classification “modified uncontrolled donors” [16].

As the lung is considered to be unique among all solid organs not relying only on perfusion to provide oxygen and cellular respiration, but air spaces, the hypothesis that lung tissue may stay viable after death has been developed. The fact that pulmonary epithelial cells have been able to be cultured from post-mortem specimens [17] have underlined that hypothesis and that even pulmonary vascular epithelium might maintain functionality for a considerable period of time. A hypothesis that epithelium of the pulmonary vasculature should even be able to produce anticoagulant factors after death cannot be sustained by any data from the literature. The tolerance of warm ischemia of the lungs and the remaining suitability of lung function has been investigated broadly. There is today a convincing amount of experimental evidence both from isolated animal lungs as well as from animal transplantation models indicating that warm ischemia of 60 min does not compromise DCD lung function and its suitability for transplantation [13–15]. The preservation technique best suitable during and after warm ischemia remains undecided. Ex vivo lung perfusion has become an important tool in assessing marginal lungs [4, 6–8, 10–12]. In DCD research, EVLP has been applied while trying to find the best suitable protocol for successful DCD lung transplantation.

While different approaches to avoiding post circulatory thrombosis in DCD have been investigated, the most beneficial preservation method is still unclear. The antegrade flush technique is well established and currently the most frequently used in clinical transplantation. Van de Wauwer and colleagues reported interesting results using a retrograde flush technique [18, 19]. They reported significantly lower PVR following their retrograde technique together with histologically less micro thrombi compared to only antegrade or non-flush techniques. Their studies were in set-ups of 60 min of warm ischemia combined with several hours of topical cooling or longer periods of post-harvest cold storage [18, 19]. More studies need to be conducted to determine whether a retrograde or a combined flush technique is superior in preventing thrombosis in DCD lungs.

The need for more ethically sensitive scenarios in DCD requires refraining from using heparin as used in standardized clinical HBD [20]. Some studies that have injected heparin [4] after declared death and applied the necessary chest compressions to the cadaver have led to conflict with the dead donor rule [21]. In order to avoid manipulating the cadaver, non-heparinized techniques or fibrinolytic agents have become more important. In earlier studies investigating the time frame of topical cooling no heparin was used by Rega et al. [22, 23], corresponding best to potential clinical scenarios of Maastricht DCD category 1 (death upon arrival) and category 2 (failed resuscitation). As heparin does not act on preformed fibrin or micro thrombi, removing the potential appearance of micro thrombi should only be possible using fibrinolytic agents. There have been reports of successful results using fibrinolytics on DCD [24–26].

In the present study, we compared no heparinized DCD lungs after 60 min of warm in-situ ischemia followed by 4 h cold storage with standard heparinized HBD lungs. Our results demonstrate a difference in blood gases when evaluating at FiO_2_ 1.0 between the standardized HBD lungs pre-treated with heparin according to clinical protocol and the no heparinized DCD lungs. The DCD group showed lower values on PaO_2_ at FiO_2_ 1.0 compared to the HBD group. Still, the PaO_2_ values of the DCD lungs fully meet the standard criteria for the acceptance for lung transplantation according to international guidelines [27]. Even the hemodynamic parameters showed equal or better results for the DCD groups. The PAF reveals significantly higher flow rates for the DCD group compared to the standardized HBD group with a PAF almost twice as high as in the HBD group. Our results for PVR were likewise in favor for the DCD group, showing statistically significant lower PVR values for DCD group than the control HBD group. These results were surprising to us, and we can only speculate that the different preservation methods between HBD and DCD might have an altering effect on the HBD lungs’ vasculature. We found prolonged time of the EVLP for HBD group compared to the DCD group. A possible hypothesis is that the significant higher flow rate (PAF) in the DCD lungs shortened the warming phase in the EVLP procedure.

Inci et al. earlier reported the use of additive urokinase in DCD to be superior over untreated DCD and HBD [25]. The long warm ischemic period in their model followed by topical cooling may have facilitated the formation and occurrence of thrombosis in the pulmonary vasculature leading to favorable results for the use of urokinase. As our findings show better results on the hemodynamics for the investigated DCD group it differs from previous study results where even topical cooling was applied after warm ischemia. It leaves the hypothesis that additional or prolonged cooling might alter the epithelium and maybe rather predispose it to formation of thrombosis. Furthermore, our tests for lung compliance post EVLP found no differences between the investigated groups. Neither did we find differences on the absence of macroscopic evidence for potential thrombi. None of the lungs in our study developed pulmonary oedema. The minimal weight gain for the DCD group of around 30 g must be seen without any clinical relevance.

Considering that a functional clinical model for Maastricht DCD category 1 and 2 could generate an increased number of grafts with good quality for transplantation, a facilitated way of treating potential donor organs is required. Retrieving organs without applying heparin will aid in that process. Less conflicting procedures on the donor body after declared death will alleviate management for staff and relatives. Cardiac chest compressions after death or even the handling of chest tubes for topically cooling the cadaver remains controversial. According to Swedish law the integrity of the body has to be respected after death until a statement of positive organ donation can be received from next of kin should no written consent exist. Any scenario involving preparative actions on the potential donor without informed consent or without a deliberative process for the relatives will evoke conflicting reactions. As this can be difficult in uncontrolled DCD settings, it should be avoided according to our opinion.

Increasing the donor pool due to facilitated procedures where donors can be left untouched for 1 h can be a promising step in meeting the present organ shortage. However, limitations in our study due to the rather short evaluation time of graft function and performance need to be considered.

## Conclusions

According to our results, DCD lungs can be safely used without heparin to achieve almost equal or results than common practice HBD lungs. Less conflicting procedures on the donor body after declared death will alleviate management for staff and relatives. The positive outcome of this animal study warrants evaluation of the method in a clinical setting.

## Abbreviations

DCD: Donation after cardiac death
HBD: Heart beating donor
EVLP: Ex vivo lung perfusion
